# How perceived school culture relates to work engagement among primary and secondary school teachers? Roles of affective empathy and job tenure

**DOI:** 10.3389/fpsyg.2022.878894

**Published:** 2022-08-11

**Authors:** Chunhua Fu, Zhen Zhao, Huimei Wang, Mingkun Ouyang, Xiaoling Mao, Xiao Cai, Xinhua Tan

**Affiliations:** ^1^School of Education, Minzu University of China, Beijing, China; ^2^School of Education Science, Guangxi Minzu University, Nanning, China; ^3^Education Center for Mental Health, Guangxi Minzu University, Nanning, China; ^4^School of Foreign Languages, Renmin University of China, Beijing, China; ^5^School of Information Science and Engineering, Yanshan University, Qinhuangdao, China

**Keywords:** job engagement, affective empathy, primary and secondary school teachers, perceived school culture, job tenure

## Abstract

Evidence suggests that perceived school culture is the most powerful predictor of teachers’ work performance. However, studies to date have paid little attention to the potential mechanisms behind this association. On the basis of the job demands–resources (JD–R) model, the present study explored the mediating role of affective empathy and the moderating role of job tenure in the association between perceived school culture and teachers’ work engagement. 647 primary and secondary school teachers completed questionnaires measuring perceived school culture, affective empathy, and work engagement. After gender and educational level were included as covariates, the results showed that perceived school culture positively correlated with teachers’ work engagement, and more importantly, this association was partially mediated by affective empathy. In addition, job tenure significantly moderated the direct association between perceived school culture and work engagement. Specifically, there was a stronger association between perceived school culture and work engagement for teachers with shorter job tenure than those with longer job tenure. The findings suggested the direct effect of perceived school culture on work engagement, and the indirect effect of perceived school culture on work engagement through the mediating role of affective empathy. These findings enrich our understanding of how perceived school culture associates with work engagement, and highlight the moderating role of job tenure in the direct association between perceived school culture and work engagement.

## Introduction

Work engagement, as one of the most studied topics, has constantly attracted attention from scholars in the field of management, psychology, organization science, and human resource development (HRD) (see reviews, Kim et al., 2013; Carasco-Saul et al., 2015; Lee et al., 2016). Work engagement is defined as “a positive, fulfilling and work-related state of mind that is characterized by vigor, dedication and absorption” (Schaufeli et al., 2002, p. 74). As a work-related factor, work engagement exerts beneficial impact on employees’ well-being, organizational commitment (Albrecht, 2012; Mazzetti et al., 2021), work performance (Kim et al., 2013; Nazarian et al., 2017; Fitria, 2018; Mazzetti et al., 2021), and career development (Lee et al., 2016). For example, greater work engagement is associated with higher job satisfaction (Granziera and Perera, 2019), and job performance (Song et al., 2018), but lower turnover intentions among teachers (Liu et al., 2017). Given the positive consequences, it is of theoretical and practical significance for HRD professionals to examine those factors which enhance employees’ work engagement (Hakanen and Roodt, 2010; Lee et al., 2016; Huang et al., 2022).

According to the job demands–resources (JD–R) model of work engagement, job and personal resources are important antecedents of work engagement (Bakker et al., 2004). Job resources are factors that help employees to deal with job demands (e.g., high workload and role ambiguity) including social- and supervisor support, developmental opportunities, and autonomy (Bakker and Demerouti, 2008; Bakker, 2015). Empirical studies showed that job resources were the most important predicator of work engagement (Crawford et al., 2010; Halbesleben, 2010; Christian et al., 2011). Job resources from working situation such as social support from colleagues, job autonomy, and performance feedback could increase employees’ work engagement, reduce job demands, achieve work goals, and stimulate personal growth and development (Bakker and Demerouti, 2007). Personal resources are defined as “those aspects of the self that are generally linked to resilience and refer to the individuals’ sense of their ability to control and impact upon their environments successfully” (Xanthopoulou et al., 2007). Examples of personal resources are optimism (Xanthopoulou et al., 2007); emotional intelligence (Mérida-López et al., 2019), and self-efficacy (Mazzetti et al., 2021). Evidence from meta-analyses showed that personal resources of personality such as optimism, conscientiousness, self-efficacy, and proactivity were positively associated with work engagement (Halbesleben, 2010; Christian et al., 2011; Mazzetti et al., 2021).

Perceived school culture as a social or organizational context factor influences teachers’ attitudes and behaviors toward work by providing information about the organizational values and norms shared by members. According to the theory of organizational culture, perceived school culture could affect teachers’ work performance and school effectiveness by influencing teachers directly or indirectly (Denision and Mishra, 1995). Recent studies established a direct association between perceived school culture and work engagement (Arifin et al., 2014; Khan, 2016; Zahed-Babelan et al., 2019; Shah et al., 2021). These findings highlighted the importance of perceived school culture in promoting teachers’ work engagement. However, the underlying mechanisms (i.e., the mediating and moderating variables) behind the association remain largely unexplored (Hartnell et al., 2019).

Haynes (2020) recently advocated using the job demands–resources (JD–R) model to explore the mechanisms underlying the relationship between perceived organizational culture and individual organizational outcomes, given that organizational culture could be considered as a sort of “second order” job resources (Schneider et al., 2017; Ancarani et al., 2019). Using the JD–R model, Haynes (2020) reported that perceived organizational culture had an indirect effect on employee turnover intentions through the mediating role of affective-motivational states (i.e., personal resources). Regarding work engagement, empirical studies provided evidence that personal resources (e.g., self-efficacy, optimism, and organizational-based self-esteem) significantly mediated the relationship between job resources (e.g., autonomy, social support, and opportunities for professional development) and work engagement (Llorens et al., 2007; Van den Broeck et al., 2008; Xanthopoulou et al., 2009). Teaching is a caring profession marked by high levels of emotional burden from the job demands. Previous research found that empathy was important in the helping and caring professions, and increased empathy could protect against stress or burnout as a result of job demands (Wilkinson et al., 2017; Williams et al., 2017). Thus, affective empathy could be well-reasoned as one of the personal resources for work engagement in teachers (Hakanen and Roodt, 2010; Stojiljković et al., 2012; Li et al., 2015). However, whether affective empathy acts as a mediating role in the relationship between perceived school culture and teachers’ work engagement remains unexplored. To address this issue, the present study aimed to examine the mediating role of affective empathy in the relationship between perceived school culture and teachers’ work engagement, based on the job demands–resources (JD–R) model that was recommended by Haynes.

In addition, although a significant relationship between perceived school culture and work engagement is empirically supported (Shah et al., 2021), little is known about the moderators of this relationship and the generalizability of the findings to other populations such as primary and secondary school teachers. Considering that job tenure (i.e., the number of years an employee has been working in the present organization) not only shaped individuals’ attitudes toward their jobs (Ng and Feldman, 2010a,b) and perceptions of the working environment (Jiang et al., 2018; Lee et al., 2019), but also significantly moderated the association between the antecedents (e.g., psychological climate and organizational culture) and work-related variables to work engagement (e.g., affective commitment) (English et al., 2010; Lee et al., 2018), it is possible that job tenure would moderate the association between perceived school culture and work engagement. Thus, the present study extends the existing research by examining the moderating effect of job tenure in the direct relationship between perceived school culture and work engagement in a sample of primary and secondary school teachers.

To sum up, this study may have contribution in three aspects. First, it may extend research on the role of potential job resources (i.e., perceived school culture) in promoting work engagement among primary and secondary school teachers, based on the JD-R model. Second, it could explain how perceived school culture is associated with work engagement through affective empathy in a mediation model. Third, it may add to previous studies by demonstrating the moderating role of job tenure in the association between perceived school culture and work engagement.

### Perceived school culture and teachers’ work engagement

Perceived school culture is defined as the values, attitudes, and behavioral norms that are created and shared by school members, including students, teachers, and other staff (Jones, 1996). According to Higgins-D’Alessandro and Sadh (1997), perceived school culture comprises two main aspects of normative expectations, i.e., interpersonal relationships and educational opportunities (e.g., justice in providing students equitable education opportunities). Based on the JD-R model of work engagement (Bakker et al., 2004), perceived school culture significantly influenced the development of student behavior and teachers’ attitude toward job (Kalkan et al., 2020). For example, research suggested that positively perceived school culture, characterized by cooperative interpersonal relationships, sharing of responsibilities, innovations, inspiring vision, and challenging mission (Brown, 2004; Družinec, 2019), promoted students’ motivation to learn, which in turn improved their academic achievement and engagement in schools (Kutsyuruba et al., 2015; Lee and Louis, 2019). By contrast, negatively perceived school culture, characterized by damaged interpersonal relationships, vague goals, lack of innovation, and inefficiency (Brown, 2004; Družinec, 2019), was associated with low achievement scores and low school engagement among students (Ripski and Gregory, 2009). As far as teachers are concerned, perceived school culture influenced teachers’ attention, how they identified with their school, work ethic, and goal achievement (Meier, 2012). Thus, it is reasonable to propose that teachers perceiving their school culture as more positive could be more engaged in their job. They may exhibit high work engagement in compliance with perceived school culture by emotionally connecting themselves to work and to others.

Some direct and indirect evidence has accumulated to support the association between perceived school culture and teachers’ work engagement. Empirical studies showed that teachers in schools with a positive school culture maintained highly motivated to teach, and were more likely to take their responsibility for students’ learning (Lee and Louis, 2019). In contrast, when teachers in a negative school culture were experiencing high levels of burnout or showing negative beliefs toward their ability to teach, both the relationships with students and the quality of their teaching practices would suffer a decrease (Skaalvik and Skaalvik, 2007). Furthermore, evidence showed that positive aspects of perceived school culture such as mutual respect and trust among school colleagues functioned as an emotional resource for sustainable school improvement (Lee and Louis, 2019). Findings particularly relevant to the present study showed that perceived school culture positively correlated with teachers’ work satisfaction (Kanesan Abdullah and Arokiasamy, 2016) and organizational commitment (Karadağ et al., 2011; Kiral and Kacar, 2016), which was positively related to work engagement (Kim et al., 2017). Taken together, the empirical evidence suggested that perceived school culture may contribute to work engagement (Arifin et al., 2014; Khan, 2016; Zahed-Babelan et al., 2019; Shah et al., 2021). Based on the theoretical and empirical grounds, we propose the hypothesis H1: Perceived school culture would be positively associated with work engagement among primary and secondary school teachers.

### Affective empathy as mediator

Affective empathy refers to the ability to perceive, share and understand another person’s affective states (Decety and Svetlova, 2012). Affective empathy is an important social–emotional capacity for successful teaching, which enables teachers to consider issues from students’ perspective and then to select optimal teaching methods in promoting students’ academic and emotional growth (Stojiljković et al., 2012; Li et al., 2015). Based on the social cognitive theory, sociocultural contexts “affect behavior through their impact on people’s sense of efficacy, aspirations, and self-regulatory factors rather than directly” (Bandura, 2002, pp. 278). Affective empathy involves a self-regulatory mechanism to separate self from other by means of modulating or exerting control over an emotional response (Decety and Jackson, 2004; Zaki, 2019). Others’ emotions may induce identical emotions in the observers through emotional contagion; the observers then needs to regulate and control those emotions in order to attend to and show concern toward others without becoming overly distressed themselves (de Waal and Preston, 2017; Stern and Cassidy, 2017). In this case, based on the social cognitive theory, affective empathy may serve as a self-regulatory factor mediating the association between perceived school culture and work engagement. To be specific, positively perceived school culture can encourage interpersonal interactions with staff members, which makes teachers more inclined to show affective empathic response to others and increase their prosocial behaviors and work engagement by offering help to those in need. Empirical evidence indirectly supported this view by showing that employee communication was considered as a mediator in the association between perceived organization culture and employee performance (Ibrahim et al., 2022).

Perceived school culture may be positively associated with teachers’ affective empathy. Affective empathy was considered as developing in the context of various social interactions where individuals learn how to regulate their own emotions as well as others’ emotions (Decety and Svetlova, 2012). School culture, as a social contextual variable, is created, shaped, and shared by school members, which in turn impacts the development of their social and emotional competence to others. Previous researches showed a positive association between perceived school culture and students’ empathy (Barr and Higgins-D’Alessandro, 2007; Schonert-Reichl et al., 2012). Compared with students, teachers were required to invest more emotional labor in teaching practices (Philipp and Schüpbach, 2010), thus their affective empathy may be also positively associated with perceived school culture. When teachers perceived a positive school culture in which they were collaboratively working with trust, cooperation, and shared responsibility, teachers could be better in empathic response to the feelings and emotional states of their students. Indeed, cross-sectional studies showed that the aspects of perceived school culture such as the quality of interpersonal relationships (Barr, 2011) and the classroom climate (O’Brennan et al., 2014) were positively associated with teacher’s empathy.

Affective empathy may be positively associated with teachers’ work engagement. According to the social neuroscience model of empathy, affective empathy involves emotion sharing and regulation, and the affective empathy is the result of the regulated emotion (Decety and Lamm, 2006). Based on this view, affective empathy could function as a protective factor against negative effects such as professional burnout by regulating one’s emotional states (Thirioux et al., 2016; Yuguero et al., 2017; Zaki, 2019). Individuals who showed higher affective empathy were more likely to report greater emotional self-efficacy (Goroshit and Hen, 2014), job satisfaction, and professional identity (Visser et al., 2018). All these issues were positively associated with higher work engagement (Granziera and Perera, 2019; Wang et al., 2020). More importantly, affective empathy was proved to be related to teachers’ job variously, for example, empathy affected social competence and communication skills (Ahmetoglu and Acar, 2016), professional identity (Glazzard and Dale, 2013; Zhu et al., 2019), and professional efficacy (Goroshit and Hen, 2016) in teachers. In other words, greater affective empathy may be positively associated with teachers’ work engagement. This view is indirectly supported by one empirical study, which confirmed that the cognitive component of empathy was positively associated with work engagement (Dal Santo et al., 2014). Given the positive roles of affective and cognitive empathy in teacher’s professional identity (Zhu et al., 2019), it is reasonable to propose that affective empathy would be positively associated with teachers’ work engagement.

Taken together, based on the empirical research mentioned, the following hypotheses is proposed: Perceived school culture would be positively associated with teachers’ affective empathy (H2a), which in turn would be positively associated with teachers’ work engagement (H2b). In other words, affective empathy would mediate the relationship between perceived school culture and teachers’ work engagement (H2).

### Job tenure as moderator

Although the direct effect of teachers’ perceived school culture on work engagement is evident in recent studies (Zahed-Babelan et al., 2019; Shah et al., 2021), this effect may not be consistent among all teachers. Therefore, it is important to explore those factors that may moderate the link between perceived school culture and work engagement. The present study proposed that job tenure would moderate the direct association between perceived school culture and work engagement.

Tenure as a proxy for work experience is defined as years in an organization (McEnrue, 1988) or at a job (McDaniel et al., 1988). According to the learning theory (March et al., 1994), people learn and develop new knowledge, skills, and abilities though work experience. Compared with employees with shorter tenure, longer tenured employees generally accumulated greater human capital over their career course (Yang et al., 2015), and had higher levels of in-role performance and citizenship behaviors (Ng and Feldman, 2010b). Additionally, employees with different job tenures had different perceptions of their work environment (Lee et al., 2019) and different capacities to manage external environments (Amirkhanyan et al., 2020). Employees with longer job tenure may be less affected by work environment because they could make optimal decisions based on their accumulated experiences and the social norms internalized. In contrast, employees with shorter job tenure must interact with others for help in their career advancement activities, thus being more sensitive to environmental conditions. To put it differently, job tenure may attenuate the effect of environmental factors, such as perceived school culture, on teachers’ work engagement (Benner, 1984).

Empirical studies provided indirect evidence for the moderating role of job tenure. One cross-sectional study showed that tenure weakened the influence of environmental factors such as authentic leadership on job satisfaction and organizational commitment among staff nurses (Baek et al., 2019). More specifically, the associations between authentic leadership and job satisfaction and between authentic leadership and organizational commitment were reported to be weaker in senior nurses than junior ones. In addition, Kiral and Kacar (2016) showed that school culture significantly predicted teachers’ commitment to their school, and this effect was more pronounced among teachers with 5 years or less job tenure than those with 11 years or more job tenure. However, there was evidence showing that job tenure strengthened the relationship between other contextual variables and work engagement. For example, studies showed that the impact of perceived high-performance work systems on affective commitment was stronger among employees with longer tenures than those with shorter tenures (Van der Westhuizen and Bezuidenhout, 2017; Hu et al., 2019). In summary, the existing studies have not yet agreed on the conclusions of how job tenure moderates the associations between environmental factors and work engagement. However, the moderating role of job tenure in these associations has been widely acknowledged. Given that school commitment and school engagement are positively intertwined concepts (Zhang et al., 2015; Kim et al., 2017), the hypothesis H3 is proposed: Job tenure would moderate the direct association between perceived school culture and work engagement. Compared with teachers with longer job tenure, those with shorter job tenure would show greater work engagement when they have the same positive perceptions of school culture.

### The present study

**FIGURE 1:**
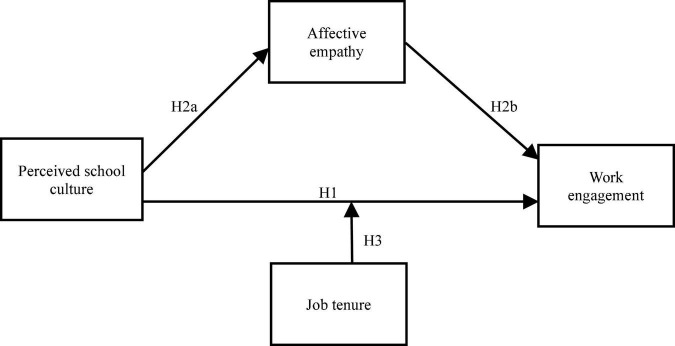
The proposed conceptual model.

Taken together, the purpose of this study is to explore the mediating and moderating mechanisms that underlying the association between perceived school culture and work engagement, in a sample of primary and secondary school teachers. Based on the previous studies, the following associations are hypothesized: (a) perceived school culture would be positively associated with work engagement; (b) perceived school culture would be positively associated with affective empathy, which in turn would be positively associated with work engagement. In other words, affective empathy would mediate the association between perceived school culture and work engagement; (c) job tenure would moderate the direct association between perceived school culture and work engagement. Specifically, the association between perceived school culture and work engagement would be stronger for teachers with shorter job tenure than those with longer job tenure. Figure 1 illustrates the proposed conceptual model.

## Materials and methods

### Participants

A total of 647 teachers, who were from primary and secondary schools in provinces of Guangdong, Anhui, and Hebei, in China, were recruited in this study using convenience sampling. Two primary and two secondary schools were selected by availability sampling in each province. About 50 teachers were selected in each school for convenience, and the number of teachers in each province was approximately two hundred. Among the recruited teachers, 9 (1.39%) of them were excluded from analyses because of out-of-range responses (e.g., writing in an unallowed number rather than selecting one of the numbers provided) and missing data. The final sample consisted of 430 females (67.4%) and 208 males (32.6%). There are 281 teachers (44.0%) with 1–10 years’ job tenure, 140 teachers (21.9%) with 11–20 years’ job tenure, 155 teachers (24.3%) with 21–30 years’ job tenure, and 62 teachers (9.7%) with more than 30 years’ job tenure. Regarding education levels, 10 teachers (1.7%) graduated from senior high school, 67 teachers (10.7%) had junior college degree, 483 teachers (75.4%) had bachelor degrees, and 78 teachers (12.2%) had master degrees.

### Measures

#### Perceived school culture

Teachers’ perceived school culture was measured by the measurement of school culture (Higgins-D’Alessandro and Sadh, 1997). The measurement consists of 25 items, and each item is rated on a *5*-point Likert scale from 1 (*not true at all*) to 5 (*very true*), with higher summed scores indexing more positive perceived school culture. A representative item is “The school attaches great importance to humanistic care, just like a big family.” This measurement showed good reliability and validity in previous study on association between school culture and teacher’s empathy (Barr, 2011). In the present study, this scale had good construct validity, χ^2^/*df* = 4.396, *p* < 0.001, RMSEA = 0.073, GFI = 0.99, TLI = 0.98, CFI = 0.99; this scale showed acceptable reliability (Cronbach’s α = 0.89).

#### Affective empathy

Affective empathy was measured by the Measure of Empathy and Sympathy, the modified version of the Adolescent Measure of Empathy and Sympathy Scale (AMES; Vossen et al., 2015; Wang et al., 2017). The subscale contains 4 items and each item is rated on a *5*- point Likert scale from 1 (*does not describe me well*) to 5 (*describes me very well*), with higher summed scores indexing higher levels of affective empathy to students. A representative item is “When my student is sad, I become sad, too.” This scale showed good reliability and validation in Chinese preschool teachers (Wang et al., 2017). In the present study, this scale had good construct validity, χ^2^/*df* = 2.544, *p* < 0.001, RMSEA = 0.049, GFI = 0.99, TLI = 0.98, CFI = 0.99; this scale demonstrated good reliability (Cronbach’s α = 0.73).

#### Work engagement

Teachers’ work engagement was measured by the shortened version of Utrecht Work Engagement Scale (UWES; Schaufeli et al., 2006). The scale consists of 9 items and each item is rated on a *7*-point Likert scale from 0 (*never*) to 6 (*always*), with higher summed scores indexing higher levels of work engagement. Items include, for example, “At my job, I feel strong and vigorous”. This scale showed good reliability and validity across different cultures (Schaufeli et al., 2006). In the present study, this scale had good construct validity, χ^2^/*df* = 4.416, *p* < 0.001, RMSEA = 0.073, GFI = 0.97, TLI = 0.96, CFI = 0.98 as well as good reliability (Cronbach’s α = 0.89).

#### Covariates

Empirical studies showed that gender moderated the association between perceived school culture and empathy (Barr and Higgins-D’Alessandro, 2007), and educational levels moderated the association between psychological contract breach and organizational outcomes (i.e., work engagement and affective commitment) (Agarwal and Bhargava, 2013). Thus, variables of gender and educational levels were included as covariates in the subsequent analyses.

### Procedure

This investigation was approved by the first author’s University Ethics Committee. Participants filled out questionnaires regarding school culture, affective empathy, and work engagement through the Questionnaire Star^1^, a professional platform for online survey, statistics, and analysis. Informed consent was obtained from participants before data collection. They were informed of anonymity and their right to withdraw from the study at any time. It took about 20 min for each to complete all the questionnaires.

### Statistical analyses

Data were analyzed using SPSS 25.0. First, we calculated descriptive statistics (i.e., *M*, *SD*) and bivariate correlation for the variables of interest. Second, we conducted a mediation analysis in PROCESS macro (Model 4; Hayes, 2017) to examine whether affective empathy mediated the relationship between perceived school culture and work engagement. Finally, we used the PROCESS macro (Model 5) to examine whether job tenure moderated the direct association between perceived school culture and work engagement. Meanwhile, we used the bias-corrected percentile bootstrap method based on 5,000 samples to calculate all indirect effects, with which 95% confidence intervals (95% *CI*s) not containing zero suggests the significance of effect at the 0.05 level. All variables of interest were standardized before being examined for the mediating and moderating effects, and covariates of gender and educational level were controlled in all analyses.

## Results

### Bivariate analyses

The descriptive statistics and Pearson correlations were presented in Table 1. Results showed that perceived school culture was positively associated with affective empathy (*r* = 0.131, *p* < 0.01) and work engagement (*r* = 0.499, *p* < 0.001); affective empathy was positively associated with work engagement (*r* = 0.219, *p* < 0.01). Thus, hypothesis 1 was supported.

**TABLE 1:** Means, standard deviations, and correlations for study variables.

	*Mean*	*SD*	1	2	3	4	5	6
(1) Gender	–	–	1					
(2) Educational level	–	–	0.025	1				
(3) Job tenure	2.000	1.044	−0.139***	−0.244***	1			
(4) Perceived school culture	18.784	4.001	−0.089*	0.048	−0.079*	1		
(5) Affective empathy	13.721	2.983	0.041	−0.107**	0.106**	0.131**	1	
(6) Work engagement	31.653	4.492	−0.116**	0.069	−0.051	0.499***	0.219**	1

* *p* < 0.05, ** *p* < 0.01, *** *p* < 0.001.

### Testing for mediation effect

**FIGURE 2:**
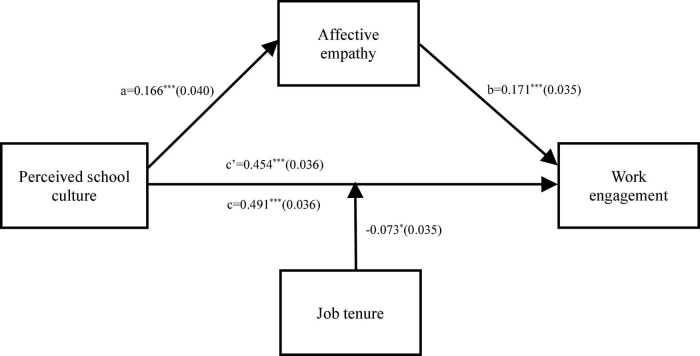
Standardized coefficients (standard errors) of perceived school culture, affective empathy, work engagement, and job tenure. *p < 0.05, ***p < 0.001.

We used Model 4 of the PROCESS macro developed by Hayes (2017) to test hypotheses 2. As shown in Table 2, perceived school culture positively associated with affective empathy (β = 0.166, *p* < 0.001) (see Model 2 of Table 2 and Figure 2), which in turn positively associated with work engagement (β = 0.171, *p* < 0.001) (see Model 3 of Table 2 and Figure 2). Thus, hypotheses 2a and 2b were supported. Meanwhile, the direct effect of perceived school culture on work engagement was also significant (β = 0.454, *p* < 0.001) (see Model 3 of Table 2 and Figure 2), suggesting that affective empathy partially mediated the association between perceived school culture and work engagement. The bootstrapping results showed that the association between perceived school culture and work engagement was mediated by affective empathy: the indirect effect = 0.028, *SE* = 0.01, *p* < 0.001, 95% *CI*s = [0.012, 0.053]. This mediation effect accounted for 5.81% (0.028/(0.028 + 0.454) × 100%) = 5.81%) of the total effect. Given that affective empathy partially mediated the association between perceived school culture and work engagement, hypothesis 2 was supported.

**TABLE 2:** Testing the mediation effect of perceived school culture on work engagement via affective empathy.

Variables	Model l (work engagement)				Model 2 (affective empathy)				Model 3 (work engagement)			
	β	*SE*	*LLCI*	*ULCI*	β	*SE*	*LLCI*	*ULCI*	β	*SE*	*LLCI*	*ULCI*
Gender	−0.152*	0.073	−0.292	−0.012	0.128	0.082	−0.033	0.289	−0.174*	0.071	−0.314	−0.034
Educational level	−0.011	0.063	−0.125	0.095	0.171*	0.071	0.031	0.311	−0.041	0.062	−0.163	0.082
Perceived school culture	0.491***	0.036	0.392	0.571	0.166***	0.040	0.087	0.245	0.454***	0.036	0.385	0.524
Affective empathy									0.171***	0.035	0.103	0.239
*R* ^2^	0.234				0.035				0.263			
*F*	64.543***				7.667***				56.345***			

β are standardized coefficients. SE, standard error; LLCI, lower limit of the 95% confidence interval; ULCI, upper limit of the 95% confidence interval. Each column is a regression model that predicts the criterion at the top of the column. * *p* < 0.05, *** *p* < 0.001.

### Testing for moderating effect

**FIGURE 3:**
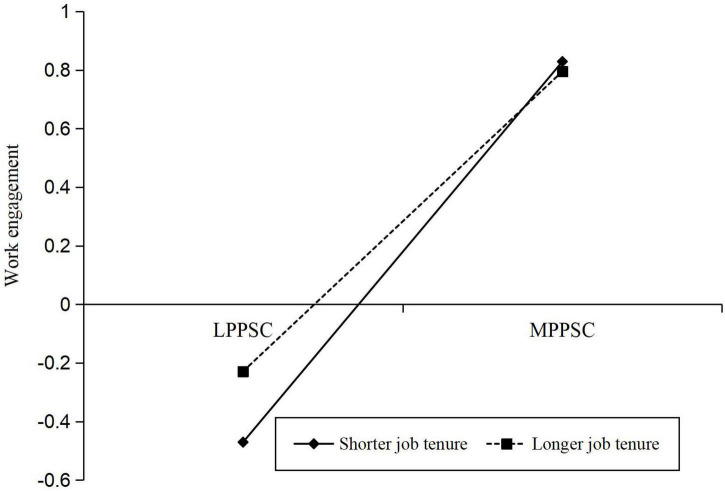
Job tenure moderates the direct relationship between perceived school culture and work engagement. LPPSC, less positive perceived school culture; MPPSC, more positive perceived school culture.

We used Model 5 of the PROCESS macro to examine whether job tenure would moderate the direct association between perceived school culture and work engagement. The results were presented in Table 3. Model 2 of Table 3 showed that perceived school culture was positively associated with work engagement (β = 0.593, *p* < 0.001), and this direct association was significantly moderated by job tenure (β = −0.073, *p* < 0.05) (see Figure 2). For clarity, we plotted perceived school culture on work engagement separately at the shorter (one standard deviation below the mean, *M − 1 SD*) and longer (one standard deviation above the mean, *M* + *1 SD*) job tenure (see Figure 3). The results of simple slope tests showed that for individuals with shorter job tenure, the positive direct effect of perceived school culture on work engagement was 0.650 (β_*simple*_ = 0.650, *t* = 6.225, *p* < 0.001); for those with longer job tenure, the positive direct effect of perceived school culture on work engagement was 0.512 (β_*simple*_ = 0.512, *t* = 11.190, *p* < 0.001) (Cohen, 1988). The association between perceived school culture and work engagement was stronger for teachers with shorter job tenure than those with longer job tenure (*Z* = 2.02, *p* < 0.05). The bias-corrected percentile bootstrap analyses also provided evidence for the moderating role of job tenure in the relationship between perceived school culture and work engagement. Specifically, the association between perceived school culture and work engagement was stronger in teachers with shorter job tenure (β = 0.512, *SE* = 0.048, 95% *CI*s = [0.418, 0.606]), but weaker for those with longer job tenure (β = 0.372, *SE* = 0.052, 95% *CI*s = [0.269, 0.475]). Thus, hypothesis 3 was supported.

**TABLE 3:** Testing the moderating role of job tenure in the mediation model.

Variables	Model 1 (affective empathy)				Model 2 (work engagement)			
	β	*SE*	*LLCI*	*ULCI*	β	*SE*	*LLCI*	*ULCI*
Gender	0.128	0.082	−0.033	0.289	−0.158*	0.072	−0.299	−0.017
Educational level	0.171*	0.071	0.031	0.311	−0.019	0.064	−0.145	0.768
Perceived school culture	0.166***	0.040	0.087	0.245	0.593***	0.073	0.446	0.741
Affective empathy					0.170***	0.035	0.102	0.238
Job tenure					0.054	0.034	−0.012	0.120
Perceived school culture*Job tenure					−0.073*	0.035	−0.141	−0.010
*R* ^2^	0.035				0.271			
*F*	7.667***				39.001***			

βare standardized coefficients. SE, standard error; LLCI, lower limit of the 95% confidence interval; ULCI, upper limit of the 95% confidence interval. Each column is a regression model that predicts the criterion at the top of the column. * *p* < 0.05, *** *p* < 0.001.

## Discussion

Although the impact of perceived school culture (as the potential job resource) on teachers’ work engagement has gained direct support (Arifin et al., 2014; Khan, 2016; Zahed-Babelan et al., 2019; Shah et al., 2021), the mechanisms underlying the association remain unknown. Based on the JD-R model and the social cognitive theory, this study formulated a mediation model to examine whether perceived school culture indirectly associated with teachers’ work engagement via the mediating role of affective empathy as the personal resource, and whether job tenure moderated the direct association between perceived school culture and work engagement. The results showed that perceived school culture was positively associated with teachers’ work engagement, and affective empathy partially mediated this relationship. In addition, job tenure moderated the direct relationship between perceived school culture and work engagement.

### The direct effect of perceived school culture on teacher’s work engagement

Consistent with hypothesis 1, the results of the present study showed that perceived school culture was positively associated with teacher’s work engagement. This finding could be explained by the JD-R model, which posits that perceived organizational culture as a potential job resource plays an intrinsic motivational role in fulfilling basic needs for relatedness, competence, and autonomy, or as an extrinsic motivational role in achieving job goals (Bakker and Demerouti, 2014). Thus, teachers perceiving positive and supportive school culture are more likely to be engaged in their job. The finding also supported the theory of organizational culture, suggesting that perceived school culture could affect teachers’ work engagement directly (Denision and Mishra, 1995). This finding was in line with the previous studies showing that perceived organizational culture was significantly associated with teachers’ organizational commitment (Karadağ et al., 2011; Kiral and Kacar, 2016; Veeriah and Siaw, 2017). Moreover, the present finding was also in line with the previous research, indicating that social context variables such as perceived organizational culture could be important predictive factors for employees’ work engagement (Shehri et al., 2017; Mushtaque and Siddiqui, 2019; Zahed-Babelan et al., 2019; Shah et al., 2021). This study illuminates the direct effect of perceived organizational culture on work engagement in a sample of primary and secondary school teachers, therefore contributing to intervention efforts toward promoting primary and secondary school teachers’ work engagement.

### The partial mediating role of affective empathy

The present study showed that affective empathy mediated the relationship between perceived school culture and teachers’ work engagement. Specifically, perceived school culture was positively associated with affective empathy, which in turn was positively associated with work engagement. Therefore, the affective empathy could serve as one of the explanatory factors for how perceived school culture contributes to teachers’ work engagement. This is the first study, to the best of our knowledge, to examine the mediating role of affective empathy in the relationship between perceived school culture and teachers’ work engagement, which was consistent with the previous studies reporting the mediating role of affective empathy (del Carmen Pérez-Fuentes et al., 2020; Schoeps et al., 2020; Chen et al., 2021). These finding illuminated how positive perceived school culture could promote teachers’ work engagement. Moreover, from a broader view, this study reveals how social-cultural context like perceived school culture is influencing teachers’ work performance.

This finding provides supporting evidence for the model of JD-R, which posits that job resources (e.g., perceived organizational culture) and personal resources (e.g., optimism, conscientiousness, and self-efficacy) available positively predict work engagement when employee are confronted with high work challenges (Xanthopoulou et al., 2007, 2009). In the previous studies on work engagement, researchers relatively neglected the possible association between job resources and personal resources (Mazzetti et al., 2020); Xanthopoulou et al. (2007) innovatively integrated resources from both fields and further considered the indirect effect of job resources on work engagement via the personal resources. It was found that personal resources significantly mediated the relationship between job resources and work engagement. The present finding adds to the previous studies by uncovering the mechanism that teachers, who perceived more positive school culture, were more likely to develop higher levels of affective empathy, which in turn led to higher levels of work engagement. The finding of mediation provided evidence for the social cognitive theory, which posits that sociocultural contexts affect behavior through their impact on people’s affective self-regulatory factors (Bandura, 2002). That is, perceived school culture may partially exert its influence on teachers’ work engagement through increasing teachers’ social–emotional capacity (i.e., affective empathy) to others.

In addition to the overall partial mediation result, each of the separate links in the mediation model is noteworthy. For the first link of the mediation process (i.e., school culture → affective empathy), positive perceived school culture was positively associated with teachers’ affective empathy. This finding was consistent with the previous studies among students (Barr and Higgins-D’Alessandro, 2007; Schonert-Reichl et al., 2012) and extends the literature by investigating the effect in a sample of teachers. This result may offer an explanation why teachers who perceive more positive school culture are better to understand and respond appropriately to their students, thereby developing higher levels of affective empathy. In other words, perceived school culture as a potential job resource fosters the development of personal resources such as affective empathy.

For the second link of the mediation process (i.e., affective empathy → work engagement), affective empathy was positively associated with teachers’ work engagement. This finding was consistent with the social neuroscience model of empathy (Decety and Lamm, 2006) and the previous studies indicating that empathy was a protective factor of job burnout (Thirioux et al., 2016; Yuguero et al., 2017; Hicks and Hanes, 2018; Zaki, 2019). One possible explanation for the present finding was that teachers with high levels of affective empathy may experience more feelings of care, concern, and compassion toward students in education (Davis, 1994), thus rendering them more likely to engage in education activities. Another possible explanation was that teachers high in affective empathy may possess high professional efficacy in dealing with stressful working conditions, thus being more willing to invest emotional efforts in their job (Goroshit and Hen, 2016).

### The moderating role of job tenure

This study showed that tenure significantly moderated the direct relationship between perceived school culture and teachers’ work engagement. Specifically, the effect of perceived school culture on work engagement was stronger for teachers with short rather than long tenure in education. This finding could be explained by the learning theory (March et al., 1994). Based on this model, longer tenured employees usually held more valuable knowledge of resources (Kang and Kim, 2019), which enhanced their abilities to deal with job tasks. Previous studies also indicated that employees with longer tenure were better to use psychological resources (e.g., self-regulatory resources) rather than job resources (e.g., organizational culture) to mitigate the work-related burnout such as job content plateau (Jiang et al., 2018). With respect to teachers, studies showed that when teachers were newcomers, their work engagement was predicted primarily by job resources offered by their school (Boswell et al., 2005; Bakker and Demerouti, 2007; Lee et al., 2011), and thus they were more likely to be affected by workplace variables such as perceived school culture. Therefore, the moderating effect of job tenure weakened the direct effect of job resources such as perceived school culture on work engagement. In this regard, teachers with short job tenure would particularly benefit from intervention programs aiming to promote positive school culture.

The results of the present study were consistent with some studies (Kiral and Kacar, 2016; Baek et al., 2019) but also inconsistent with other studies (Van der Westhuizen and Bezuidenhout, 2017; Hu et al., 2019). These inconsistent findings may be partly due to the differences in sample characteristics. For example, Hu et al. (2019) used a sample of employees from an information technology company, while the present study used teachers of primary and secondary schools. Compared to teachers in educational setting, employees’ role behavior and organizational citizenship behavior in company may be timely recognized and rewarded by managers, which could motivate them to engage to their work, and especially those with longer organizational tenure (Liao et al., 2009). This finding suggested that when employees in an organization perceive plenty of supportive organizational culture fitting their career development, their work engagement would be more easily influenced by organizational culture.

Overall, by integrating job tenure as a moderator into the mediation model, this study uncovered effects that may have been neglected without the moderation analysis. The mediation model with moderation in the present study is conceptually more nuanced and provides greater predictive power than the mediation model or moderation model alone.

### Limitations and implications

Limitations should be addressed in future study. Firstly, cross-sectional data in the present study limited drawing causal inferences regarding the associations among the variables of perceived school culture, affective empathy, and work engagement. Additionally, cross-sectional data are prone to generate biased estimates of mediation effects, and the terms of “mediation/mediated” used in the study may lead to false faith that perceived school culture positively influenced affective empathy, which in turn would promote teacher’s work engagement (Maxwell and Cole, 2007). Future studies should use longitudinal or experimental designs to further examine our mediation model. Secondly, findings in the present study were based on the convenience sample of primary and secondary school teachers, limiting the generalizability to other samples. Thirdly, self-report measures used to collect data are susceptible to method bias, although Harman’s one-factor test in the present study showed that the first factor only accounted for 37.35% of the total variance, which was less than the critical value of 40% (Podsakoff and Organ, 1986). Multiple measures such as in-depth interview and behavioral observations in daily life should be used in future research.

Despite these limitations, our findings have important theoretical and practical implications. From a theoretical perspective, the present study provides an empirical framework of how and when perceived school culture relates to work engagement, which deepens our understanding of the mechanisms underlying teachers’ work engagement. From a practical perspective, the current findings are critical for interventions aimed at increasing teachers’ work engagement. First, a positive perceived school culture should be created to foster teachers’ work engagement. School administrators should develop sincere and honest relationships with school members, and transform the school as a sustainable institution into a learning organization with the participation of all school members (Kalkan et al., 2020). Second, given that affective empathy acts as a mediating role in the relation between perceived school culture and work engagement, empathy-based interventions should be developed to increase teacher’ affective empathy. Studies confirmed that the development of empathy ability of teachers could be achieved through empathy training in different ways (Crichton and Carter, 2016). For example, a virtual reality (VR)-based training program fostered the development of empathy skills for teachers (Stavroulia et al., 2018). Besides, psychological interventions though improving empathic motivation such as changing the theories of students’ development could be adopted to cultivate empathy for teachers in education training programs (Ge et al., 2021).

Third, although perceived school culture is positively associated with teachers’ work engagement, the direct effect differed for teachers with diverse years of tenure in teaching. It is helpful to realize that teachers with longer years of tenure in teaching are less sensitive to the influences of school culture on their work engagement. Previous studies showed that job demands and job resources interacted in predicting work engagement, i.e., job resources exert the strongest positive influence on work engagement when job demands were challenging (Bakker et al., 2007). Thus, we are supported to provide tenured teachers with challenging job demand in amplifying the positive impact of job resources on work engagement. However, for teachers with shorter job tenure, efforts to create a positive perceived school culture will boost individuals’ work engagement during the interaction with students.

## Conclusion

In summary, the present study showed that perceived school culture was positively associated with primary and secondary school teachers’ work engagement. Furthermore, mediation analysis indicated that affective empathy partially mediated the association between perceived school culture and teachers’ work engagement. In addition, moderation analysis showed that job tenure moderated the direct relationship between perceived school culture and teachers’ work engagement, with the effect being stronger for teachers with shorter job tenure.

## Data availability statement

The original contributions presented in this study are included in the article/supplementary material, further inquiries can be directed to the corresponding authors.

## Ethics statement

The studies involving human participants were reviewed and approved by the Research Ethics Committee of Minzu University of China. The patients/participants provided their written informed consent to participate in this study.

## Author contributions

CF, ZZ, and HW: conceptualization, software, and writing – original draft. MO and XM: writing, supervision and validation. XC and XT: review and editing. All authors contributed to the article and approved the submitted version.

## Footnotes

1
http://www.wjx.cn
